# Positive and Negative Emotional Eating Are Not the Same—The Spanish Version of the Positive-Negative Emotional Eating Scale (PNEES)

**DOI:** 10.3389/fpsyg.2021.709570

**Published:** 2021-07-05

**Authors:** Javier Manchón, María José Quiles, Yolanda Quiles, Sofía López-Roig

**Affiliations:** Miguel Hernández University of Elche, Alicante, Spain

**Keywords:** positive emotional eating, negative emotional eating, validation, eating disorders, overeating, binge eating

## Abstract

The literature points to the importance of distinguishing between positive and negative emotional eating in relation to overeating and binge eating. The aim of this study was to evaluate the Spanish version of the Positive-Negative Emotional Eating Scale (PNEES) in a Spanish community sample. The sample consisted of 628 participants. The mean age was 27.5 (*SD* = 12.7) and 70.1% of them were women. The participants completed the PNEES, and measures of anxiety and depression (HADS), and eating disorder-related scales (TFEQ-R18, BULIT-R, and EAT-26) that were selected to examine convergent validity. A confirmatory factor analysis was conducted, replicating the original two-factor solution, consisting of Negative Emotional Eating (PNEES-N) and Positive Emotional Eating (PNEES-P). The results showed an acceptable fit of the model (CFI = 0.986; TLI = 0.984, RMSEA = 0.055). Internal consistency ranged from ω = 0.92 to ω = 0.96 for both subscales and the total score. PNEES-P correlations with other variables were lower with respect to the PNEES-N, showing that they are different constructs. A mediation analysis was conducted, in which PNEES-P significantly predicted binge eating and PNEES-N was a partially mediator variable. The results showed that the adaptation process was successful.

## Introduction

The concept of emotional eating stems from psychosomatic theory (Bruch, 1964) which proposes that emotional eaters are unable to distinguish hunger from the physiological state accompanying negative emotions. In this way, as Adriaanse et al. (2011) explain, emotional eating occurs in response to experiencing negative emotions, whereas ordinarily these emotions would result in loss of appetite since emotions induce physiological changes similar to those of satiety.

Macht (2008) specified five classes of emotion-induced changes of eating: (1) emotional control of food choice, (2) emotional suppression of food intake, (3) impairment of cognitive eating controls, (4) eating to regulate emotions, and (5) emotion-congruent modulation of eating. This author concluded that emotion-induced changes of eating can be a result of interference of eating by emotions, a by-product of emotions, and a consequence of regulatory processes (i.e., emotions may regulate eating, and eating may regulate emotions).

Despite this classification, emotional eating has traditionally been associated with the tendency to increase one’s food intake in response to negative emotions, such as anxiety, depression, anger or loneliness. Emotional eating theory states that negative emotions can induce eating, because eating has the capacity to reduce their intensity (Macht and Simons, 2011). Emotional eating has been associated with binge eating, overeating, bulimia nervosa and obesity (Arnow et al., 1992; Faith et al., 1997; Fox and Power, 2009). A recent meta-analysis, consisting of 56 experimental studies, assessed the causal effect of emotions on eating in both healthy and eating disordered individuals. Results showed that restrained eaters showed increased eating in response to negative emotions. Negative emotions did not affect eating in overweight or obese people, people with eating disorders, or self-assessed emotional eaters. Positive emotion resulted in increased eating across groups (Evers et al., 2018). Similar results were obtained in a meta-analysis of laboratory studies aimed at assessing the effect of induced negative and positive mood on meal consumption in healthy participants and patients with eating and weight disorders (Cardi et al., 2015). Induced negative mood was significantly associated with greater food intake, especially in restrained eaters and binge eaters. Positive mood was also associated with greater caloric intake across groups.

These studies highlight the importance of positive emotions in emotional eating behavior. So far, different instruments have been developed to assess emotional eating. The first questionnaires that were used to assess emotional eating were not created specifically for this purpose, and moreover, they were focused mainly on its relation to negative emotions, leaving positive emotions out of consideration. These include The Three Factor Eating Questionnaire (TFEQ; Stunkard and Messick, 1985), with three scales assessing Disinhibition, Dietary Restraint and Perceived Hunger, and The Dutch Eating Behavior Questionnaire (DEBQ; van Strien et al., 1986) which contains three scales; emotional eating, externality and restraint. Both questionnaires have been criticized for failing to provide insight into the relationship between specific mood states (e.g., anger, anxiety and depression) and overeating (Arnow et al., 1995; Sultson et al., 2017).

Subsequently, Arnow et al. (1995) developed the Emotional Eating Scale, a specific questionnaire to assess emotional eating and that would provide a more detailed analysis of the relationship between negative moods and disordered eating. The questionnaire consisted of 25 items distributed in three subscales; Anger/Frustration, Anxiety, and Depression.

All these questionnaires have only focused on negative emotions, however, it is also important to know how positive emotions relate to overeating and binge eating. The Emotional Appetite Questionnaire (EMAQ; Geliebter and Aversa, 2003; Nolan et al., 2010) was developed with this aim. The EMAQ contains 22 questions about the tendency to eat in response to positive and negative emotions (14 items) and to positive and negative situations (8 items). The positive emotion (EMAQ-PE) and positive situation (EMAQ-PS) scores can be averaged to obtain a positive EMAQ score (EMAQ-P). The negative emotion (EMAQ-NE) and negative situation (EMAQ-NS) scores can also be averaged to obtain a negative EMAQ score (EMAQ-N). The EMAQ has demonstrated high test-retest reliability (r coefficients ranged from 0.71 to 0.95) and acceptable internal consistency ranging from 0.57 to 0.78 (Geliebter and Aversa, 2003).

Another questionnaire that includes the assessment of both negative and positive emotions is the *Positive-Negative Emotional Eating Scale* (PNEES; Sultson et al., 2017). The interest of this questionnaire is that it measures the tendency to eat in response to specific positive and negative emotions. With respect to its psychometric properties, the PNEES is made up of 19 items that loaded in two factors, the Negative Emotional Eating (PNEES-N) and the Positive Emotional Eating (PNEES-P). The PNEES-N factor accounted for 40.1% of total variance and the PNEES-P for the 22.9%. The PNEES showed good internal consistencies for the total score and subscales, and acceptable fit of the two-factor model. PNEES-N showed good convergent validity in assessing binge eating. Positive emotional eating significantly predicted binge eating, even though the effect was partly mediated by negative emotional eating. Therefore, PNEES-P predicted Binge eating regardless of PNEES-N.

Another distinctive feature of the PNEES is that it reflects real-life eating behavior. This scale showed its predictive validity on overeating and binge eating episodes measured via ecological momentary assessment, which involves repeated measurements of individuals’ current behaviors and experiences in real time and in natural environments. In this questionnaire, negative emotional eating predicted overeating and binge eating episodes in women who experienced at least one overeating or binge eating episode (Sultson et al., 2017).

Currently, there are no self-report questionnaires validated in a Spanish population to measure how negative and positive emotions are related to overeating and binge eating. This study provides the first translation and evaluation of a Spanish version of the PNEES. Thus, the aim of the present study was to evaluate the Spanish version of the PNEES in a Spanish community sample. For this purpose, (1) we used confirmatory factor analysis (CFA) to test the factor structure proposed by the authors of the original version, (2) we checked the internal consistency as well as the convergent and discriminant validity, and (3) we conducted a mediation analysis to test the scale’s concurrent validity.

## Methods

### Sample

The sample consisted of 628 participants from a community sample. The mean age was 27.53 (*SD* = 12.70) and 70.1% (*n* = 440) of them were women. Their Body Mass Index (BMI) was calculated from self-reported weight and height. Their mean BMI was 22.90 (*SD* = 3.90). With respect to their weight status, 9.3% of the participants were underweight, 67.8% of them presented normal weight 18.3% were overweight 4.6% of them had obesity. Regarding the highest level of education completed, 16 participants reported that they completed primary education (2.5%), 460 of them completed secondary education (73.2%), 106 of them completed university degrees (16.9%) and 46 of them completed postgraduate or doctoral studies (7.3%).

### Procedure

The project and the data collection were approved by the Research Ethics Committee of the university.

The translation and cultural adaptation procedure was based on the study by López-Roig and Pastor (2016). Two authors translated the 19 items independently. The items were compared to guarantee a correct translation into Spanish. Later, back-translation was performed by two bilingual speakers (Spanish and English). Their translations were compared in order to resolve disagreements between versions.

The questionnaires were administered online. Sampling was carried out by sharing a link on social networks (WhatsApp, Instagram, and email) to general population, for them to fill it out and send it to their families or friend to fill it out as well. Before starting, the participant information sheet and the consent form were available to the participants. If they agreed to participate, they signed the informed consent and completed the questionnaires. No economic compensation of any kind was offered.

### Outcome Measures

*Positive and Negative Emotional Eating Scale* (PNEES; Sultson et al., 2017). The original version of this scale has 19 items measuring the tendency to eat in reaction to positive (PNEES-P, 7 items) or negative (PNEES-N, 12 items) emotions. The items were constructed following the emotions included in the Positive and Negative Affect Schedule (PANAS; Watson et al., 1988). It consists of a Likert-type scale ranging from 0 (never) to 4 (very often). In the original study, internal consistency was adequate across the two factors (α = 0.95 for PNEES-N and α = 0.91 for PNEES-P) and the overall score (α = 0.93). PNEES has also shown evidence of construct validity, as it moderately correlated with restrained eating, purging (only PNEES-N), preoccupation with body image and body weight, and binge eating. PNEES-N appeared to partially mediate the effect of PNEES-P on binge eating.

*Hospital Anxiety and Depression Scale* (HADS; Zigmond and Snaith, 1983; Spanish version by Terol-Cantero et al., 2007). This scale consists of 14 items that measure anxiety and depression with seven items each. The item scores range from 0 to 3 points. Higher scores suggest higher anxiety and depression. Internal consistency of the Spanish version ranged from 0.69 to 0.75. In this study, the internal consistency values were α = 0.78 for the total score, α = 0.72 for anxiety and α = 0.83 for depression.

*The Three-Factor Eating Questionnaire* (TFEQ-R18; Stunkard and Messick, 1985; Spanish version by Jáuregui-Lobera et al., 2014). It measures three factors of eating behaviors, these being cognitive restraint, uncontrolled eating and emotional eating. The revised version consists of 18 items with a Likert-scale from 1 (Completely true) to 4 (Completely false). Higher scores on the corresponding scale mean more cognitive restraint, more uncontrolled eating and more emotional eating. The Spanish version showed adequate internal consistency (α = 0.83). In this study, the internal consistency was α = 0.88.

*Binge Eating subscale from Bulimia Test-Revised* (BULIT-R; Thelen et al., 1991; Spanish version by Mora and Raich, 1993). This questionnaire was designed to detect the risk of developing bulimia. For this study, only the 14 items of the binge eating subscale were administered. The items are answered on a five-point Likert scale, with several options for each question. Internal consistency of the questionnaire ranges from 0.92 to 0.98. In this study, the internal consistency of the subscale was α = 0.87.

*Eating Attitudes Test* (EAT-26; Garner et al., 1982; Spanish version by Gandarillas et al., 2002). This self-report instrument is used as a screening measure for the detection of cases of eating disorders. It consists of 26 items grouped into three subscales: Dieting, bulimia, and food preoccupation and oral control. It is measured with a Likert-scale of six points from Never to Always. Internal consistency for all factors were α = 0.88 (Dieting), α = 0.77 (Bulimia) and α = 0.58 (Oral control). In this study, internal consistency values were α = 0.85 (Dieting), α = 0.78 (Bulimia) and α = 0.66 (Oral control).

### Statistical Analyses

The statistical computing R environment 4.0.1 was used for the data analyses. First, the CFA was conducted with R’s lavaan package (Rosseel, 2012). Following the conclusions from Li (2015), the method of parameter estimation was DWLS (diagonally weighted least squares) since data were ordinal and non-normally distributed. The indices used for testing the model fit were the chi-square test, the comparative fit index (CFI), the Tucker-Lewis index (TLI) the root mean square error of approximation (RMSEA) and the standardized root mean-square (SRMS). R’s psych package (Revelle, 2020) was used to perform internal consistency analyses (α and ω coefficients), Pearson correlations and mediational analyses.

## Results

### Factor Structure

First, the CFA was performed with the DWLS estimation. A two-factor solution replicating the original study was tested. Therefore, 12 items were allocated in the Negative Emotional Eating factor and seven items were allocated in the Positive Emotional Eating factor. The results showed an acceptable fit of the model [χ^2^_(__151__)_ = 441.87, *p* < 0.01; CFI = 0.986; TLI = 0.984, RMSEA = 0.055 (90% CI:0.049 ∼0.061), SRMR = 0.064]. All parameter estimates were significant and above 0.70 in both factors. PNEES-N and PNESS-P were also correlated (*r* = 0.33, *p* < 0.01). The parameter estimates are presented in Table 1.

**TABLE 1:** Parameter estimates of the two factors.

**Items**	**PNEES-N**	**PNEES-P**
1. Grumpy (malhumorado/a)	0.849	
2. Upset (alterado/a)	0.848	
3. Active (activo/a)		0.704
4. Disappointed (decepcionado/a)	0.827	
5. Joyful (alegre)		0.814
6. Irritated (irritado/a)	0.840	
7. Tense or anxious (tenso/a o ansioso/a)	0.790	
8. Sad (triste)	0.776	
9. Full of energy (lleno/a de energía)		0.760
10. Content with myself (satisfecho/a conmigo mismo/a)		0.793
11. Helpless (indefenso/a)	0.789	
12. Restless (inquieto/a)	0.778	
13. Offended (ofendido/a)	0.753	
14. Excited about something (entusiasmado/a por algo)		0.888
15. Angry (enfadado/a)	0.812	
16. Lonely (solo/a)	0.790	
17. Confident (seguro/a de mí mismo/a)		0.778
18. Feeling guilty (culpable)	0.769	
19. Fascinated about something (fascinado/a por algo)		0.798

*Spanish translation of the adjectives is included in brackets.*

### Reliability

Cronbach’s α was calculated for PNEES-N (α = 0.96), PNEES-P (α = 0.92) and the total score (α = 0.94). Furthermore, McDonald’s ω was also calculated, yielding similar results (ω = 0.96 for PNEES-N, ω = 0.92 for PNEES-P and ω = 0.96 for total score). These results show evidence of good internal consistency and are very similar to the original scale.

### Construct Validity

Measures of anxiety, depression and eating disorder-related variables were selected to examine their relations with emotional eating. Table 2 includes the descriptive analysis of the variables as well as Pearson’s correlations with the PNEES. Negative emotional eating correlated with every variable except for Oral control (*r* = −0.09), while positive emotional eating did not correlate with Restraint (*r* = −0.06), and the three factors from EAT scale. Interestingly positive emotional eating correlations were lower with respect to the negative emotional eating factor.

**TABLE 2:** Descriptive analysis and correlations between negative and positive emotional eating and other variables.

	**M (SD)**	**Range**	**PNEES-N**	**PNEES-P**
PNEES-Negative emotional Eating	14.20 (12.32)	0–48	1	0.332**
PNEES-Positive emotional Eating	9.29 (6.78)	0–28	0.332**	1
HADS-Total	11.82 (5.45)	0–42	0.292**	0.174**
HADS-Anxiety	9.11 (3.73)	0–21	0.224**	0.146**
HADS-Depression	2.70 (3.13)	0–21	0.242**	0.130**
BULIT-Binge eating	24.44 (7.65)	14–70	0.540**	0.261**
TFEQ-Emotional eating	6.05 (2.66)	3–12	0.779**	0.186**
TFEQ-Disinhibition	19.14 (5.80)	9–36	0.582**	0.377**
TFEQ-Restraint	12.50 (4.33)	6–24	0.255**	−0.057
EAT-Diet	7.17 (7.02)	0–52	0.397**	0.048
EAT-Bulimia	1.76 (2.91)	0–24	0.483**	0.090
EAT-Oral control	3.02 (3.33)	0–32	–0.094	0.041

**p < 0.01.PNEES-P, Positive Emotional Eating; PNEES-N, Negative Emotional Eating.

Correlations between PNEES and BMI were also obtained. Negative emotional eating correlated with BMI (*r* = 0.13, *p* < 0.01), while positive emotional eating did not (*r* = −0.08, *p* > 0.05).

### Mediation Analysis

**FIGURE 1:**
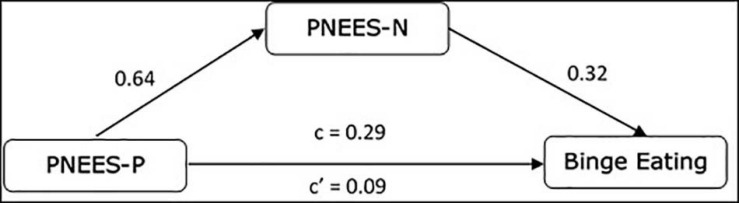
Mediational analysis. All regression coefficients were significant at least at *p* < 0.05.

A path analysis was conducted replicating the original study. As in the original study, binge eating was the dependent variable, PNEES-P acted as the independent variable and PNEES-N as the mediator. Both direct (β = 0.09, *SE* = 0.04, *p* < 0.05) and indirect (β = 0.20, *SE* = 0.03, *p* < 0.01) effects were significant. The total effect of PNEES-P on binge eating was also significant (β = 0.29, *SE* = 0.04, *p* < 0.01). These results show that PNEES-N was a partially mediator variable (Figure 1). The analysis accounted for 30% of the variance of binge eating (*R*^2^ = 0.30, *F* = 132.37, *p* < 0.01), explaining less than the path analysis of the original study of Sultson et al. (2017).

## Discussion

The objective of the present study was to translate the PNEES to Spanish and assess its psychometric properties in a sample of Spanish population. Currently, there is no other translated version of the original PNEES. The results showed that the adaptation process was successful.

First, the CFA reproduced the factor structure from the original validation. Thus, the 19 items were grouped into the subscales Positive Emotional Eating and Negative Emotional Eating and the factor loadings of every item were above 0.70, showing a strong factor structure. Moreover, PNEES-P and PNEES-N showed a moderate correlation, in line with the original study. Internal consistency was optimal for both subscales and total score. These results were very similar to those found in the original study (Sultson et al., 2017; Sultson and Akkermann, 2019).

With respect to evidence of convergent and discriminant validity, correlations with other instruments were analyzed. The results were in line with the original validation (Sultson et al., 2017) and provided additional insight. PNEES-N and PNEES-P showed a different performance. PNEES-N was highly correlated with eating disorders variables such as disinhibition (TFEQ), binge eating (BULIT), and bulimia (EAT-26), while it was moderately correlated with anxiety and depression (HADS), restraint (TFEQ), and dieting (EAT-26). This was not the case for PNEES-P, as the correlations were lower in all cases. It was moderately correlated with binge eating and disinhibition and slightly correlated with anxiety and depression. Moreover, the TFEQ factor emotional eating only showed strong correlation with PNEES-N, and PNEES-N and PNEES-P were moderately correlated with each other. Altogether, these results suggest that positive and negative emotional eating are two distinct variables, the latter of which relates more to maladaptive eating behaviors.

Looking specifically at some of these relationships, it can be noted that negative emotional eating was related to a negative emotional state (HADS scores). Previous research has shown that anxiety (Forney et al., 2016) and depression (Goldschmidt et al., 2012) can influence the amount of food eaten in a binge eating episode. Negative emotional eating, then, might serve as a regulation strategy in such a way that individuals engage in overeating because of their inability to implement more adaptive regulation strategies (Whiteside et al., 2007). For the positive emotional eating subscale, correlations were found with binge eating and disinhibition as mentioned above. Previous studies have also associated positive emotional eating with overeating and binge eating (Bongers et al., 2016). Positive emotions are related to an increased drive to eat, especially highly palatable and sweet foods (Cardi et al., 2015). In this sense, positive emotional eating emphasizes that eating is not only used as a strategy to manage negative emotions but can also be used to intensify a pleasant emotional state.

It is important to note that negative emotional eating had a partial mediating effect between positive emotional eating and binge eating. Although in both the original study and this paper negative emotional eating was a partial mediator, the regression coefficients varied. While in Sultson et al. (2017) work the clearest relationship was the explanation of negative emotional eating toward binge eating, in our case the strongest relationship was found between positive and negative emotional eating. To our point of view, these differences may be due to sociodemographic differences in the samples, since the authors of the original paper assessed the questionnaire only in women. The literature shows that emotional eating is more frequent in women than in men (Tan and Chow, 2014; Verzijl et al., 2018), a fact that could be influencing the observed relationships.

Nevertheless, this result suggests that positive emotions may also be a predictor of binge eating. As mentioned above, binge eating is often associated with negative mood (Berg et al., 2015). People experiencing a negative mood state who overeat tend to consume palatable foods and high density food and this may lead to a corresponding weight gain (Devonport et al., 2019; Al-Musharaf, 2020). However, the positive emotional eating has been neglected in the literature. Therefore, we consider it important that positive emotional eating should be included in future eating behavior research and should be targeted in interventions related to eating behavior, such as binge eating disorder and bulimia, but also weight loss interventions for people with obesity.

There are several limitations that should be noted. Regarding the sample, although the number of participants was adequate for the length of the questionnaire, the sample was community-based and mostly composed of women. Further research is needed with larger samples and a greater recruitment of men. Future studies should also include a clinical sample in order to assess the PNEES properties in this kind of population. It would also be interesting to assess the questionnaire’s sensitivity to change to a clinical intervention. Test–retest reliability has not been assessed in this study and the predictive validity of this instrument should be assessed in future research.

## Data Availability Statement

The datasets presented in this article are not readily available because participants of this study did not agree for their data to be shared publicly, so supporting data is not available. Requests to access the datasets should be directed to MQ.

## Ethics Statement

The studies involving human participants were reviewed and approved by Oficina de Investigación Responsable from Miguel Hernández University of Elche. The patients/participants provided their written informed consent to participate in this study.

## Author Contributions

JM, MQ, and SL-R designed the study and carried out the process of adaptation into Spanish. JM, MQ, and YQ collected the data and wrote the manuscript. JM analyzed the data. SL-R gave feedback on the manuscript. All authors approved the final version.

## Conflict of Interest

The authors declare that the research was conducted in the absence of any commercial or financial relationships that could be construed as a potential conflict of interest.
